# Long-term MODIS observations of cyanobacterial dynamics in Lake Taihu: Responses to nutrient enrichment and meteorological factors

**DOI:** 10.1038/srep40326

**Published:** 2017-01-11

**Authors:** Kun Shi, Yunlin Zhang, Yongqiang Zhou, Xiaohan Liu, Guangwei Zhu, Boqiang Qin, Guang Gao

**Affiliations:** 1 Taihu Laboratory for Lake Ecosystem Research, State Key Laboratory of Lake Science and Environment, Nanjing Institute of Geography and Limnology, Chinese Academy of Sciences, Nanjing 210008, China; 2 State key Laboratory of Satellite Ocean Environment Dynamics, Second Institute of Oceanography, State Oceanic Administration (SOA), People’s Republic of China, Hangzhou 310012, China; 3 University of Chinese Academy of Sciences, Beijing 100049, China

## Abstract

We developed and validated an empirical model for estimating chlorophyll *a* concentrations (Chl*a*) in Lake Taihu to generate a long-term Chl*a* and algal bloom area time series from MODIS-Aqua observations for 2003 to 2013. Then, based on the long-term time series data, we quantified the responses of cyanobacterial dynamics to nutrient enrichment and climatic conditions. Chl*a* showed substantial spatial and temporal variability. In addition, the annual mean cyanobacterial surface bloom area exhibited an increasing trend across the entire lake from 2003 to 2013, with the exception of 2006 and 2007. High air temperature and phosphorus levels in the spring can prompt cyanobacterial growth, and low wind speeds and low atmospheric pressure levels favor cyanobacterial surface bloom formation. The sensitivity of cyanobacterial dynamics to climatic conditions was found to vary by region. Our results indicate that temperature is the most important factor controlling Chl*a* inter-annual variability followed by phosphorus and that air pressure is the most important factor controlling cyanobacterial surface bloom formation followed by wind speeds in Lake Taihu.

Lakes deliver services of enormous global value by acting as indicators and regulators of climate change and by providing crucial resources for humans^1^,^2^. However, as human activities and economic development have increased over the last few decades, lake eutrophication has become a serious ecological, environmental and social problem in both industrialized and developing countries^3^,^4^,^5^. Among human-induced changes to lake environments, nutrient over-enrichment (especially of nitrogen and phosphorus) associated with urban, agricultural and industrial development is one of the most notable. This has led to accelerated rates of lake eutrophication^6^. Lake eutrophication can result in an increase in primary production, which in turn spurs the development of larger and more frequent cyanobacterial blooms^7^,^8^. Around the world, numerous eutrophic lakes suffer from severe cyanobacterial blooms, including Lake Taihu^9^, Lake Chaohu^10^ and Lake Dianchi^11^ in China; Lake Erie in the U.S. and Canada^5^; Lake Winnipeg in Canada^12^; and Lake Nieuwe Meer in the Netherlands^13^.

Cyanobacterial blooms can severely stress the ecological structures and functions and aesthetics of lake ecosystems^5^,^14^. Large populations of cyanobacteria can increase lake turbidity levels due to high chlorophyll *a* concentrations (Chl*a*) present at the water surface, thereby decreasing light penetration levels^15^. In turn, less light is available for submerged aquatic vegetation (SAV), thus suppressing SAV growth and populations^16^,^17^. Moreover, severe cyanobacterial blooms can lead to dissolved oxygen depletion at night, in turn decreasing fish populations^18^. Some cyanobacterial species are known to produce a variety of toxins (toxic peptides and alkaloids)^19^,^20^; the ingestion of such cyanotoxins can have serious effects on animal and human health (i.e., liver, digestive and skin diseases; neurological impairment and death)^21^,^22^. Therefore, cyanobacterial blooms pose a major threat to the use of lake ecosystems for drinking and irrigation water, fishing and recreational purposes^15^,^20^,^23^.

To manage, control, and treat cyanobacterial blooms, it is crucial to accurately quantify cyanobacterial bloom responses to climate change and nutrients and to identify the mechanisms of these responses. Many recent studies have used field and laboratory data and simulation models to quantitatively and qualitatively assess the role of climate change and nutrients in cyanobacterial surface bloom expansion in inland, coastal and ocean waters^4^,^6^,^23^,^24^,^25^,^26^,^27^,^28^,^29^. However, many of these studies introduce a substantial degree of uncertainty due to the complexities and costs of *in situ* measurements of cyanobacterial abundance and difficulties associated with generalizing laboratory findings over large spatial and temporal scales^30^,^31^. These complex processes interact to produce physical, chemical, and biotic perturbations of lake ecosystem functioning, thus raising the need for new quantitative methods for the detection, quantification, synthesizing and modeling of impacts at broad spatial and temporal resolutions^17^.

The spatial and temporal coverage provided by satellite remote sensing makes it an attractive tool to use in such studies. Remotely sensed data such as those generated by the Moderate Resolution Imaging Spectroradiometer (MODIS; from the Aqua satellite for 2002-the present) provide opportunities to quantify cyanobacterial biomass and production levels in lake and ocean waters at a fine spatial and temporal resolution. Satellite observations are critical to identifying and removing spatial and temporal aliasing from scarce field measurements when investigating long-term baseline information and trends of cyanobacterial production. To develop a stronger understanding of the effects of human activities and climate change on cyanobacterial blooms, Paerl and Huisman (2009) recommended coupling traditional sampling and remote sensing methods to monitor cyanobacteria-dominated aquatic ecosystems^8^. Several recent studies have successfully applied long-term remotely sensed data to model the responses of phytoplankton production dynamics to environmental variables in coastal and ocean waters^31^,^32^,^33^,^34^,^35^. For example, Vope *et al*. (2012) used MODIS-derived Chl*a* data for the Mediterranean Sea to establish that phytoplankton populations were responding to physical variables such as sea surface temperatures^35^, thus identifying mechanisms that link Chl*a* and sea surface temperature. Keith (2014) found a relationship between Chl*a* variations and nutrients in the Neuse and Tar-Pamlico River estuaries, demonstrating that the remote sensing of Chl*a* can support nutrient management^32^. One widely used proxy for cyanobacterial biomass monitoring is the Chl*a*^3^,^14^,^36^ for inland cyanobacteria-dominated aquatic ecosystems.

Remote sensing techniques have rarely been applied to quantify the responses of cyanobacterial biomass dynamics to climate change and to nutrient variations for inland lake waters. This is partly attributable to the fact that it is challenging to accurately derive Chl*a* (an index of cyanobacterial biomass) values from satellite measurements of optically complex cyanobacteria-dominated inland lakes due to the presence of non-covarying optically active constituents such as colored dissolved matter (CDOM) and organic and inorganic suspended matter^37^. Several algorithms have been presented that use red to near-infrared bands to estimate Chl*a* values for inland lake waters. These include (1) two-band empirical algorithms (Moses *et al*. 2009); (2) three- and four-band empirical algorithms^36^,^38^,^39^,^40^; and (3) maximum peak height (MPH), fluorescence line height (FLH), and maximum chlorophyll index (MCI) algorithms^26^. Among the satellite sensors with high spatial and temporal resolutions, only the MERIS can generate appropriate data for red to near-infrared band algorithms. MERIS imagery offers a fine spatial (full resolution: 300 m) and spectral resolution and a short revisit time (near daily coverage) and was therefore believed to serve as an ideal satellite image dataset for monitoring Chl*a* in inland lake waters. Unfortunately, the MERIS sensor was discontinued in April of 2012. As an alternative, the MODIS-Aqua sensor was launched in May of 2002; by offering a short revisit time (one day) and relatively high spatial resolution (250 m for the first two bands), it can generate data of a sufficient spatial and temporal resolution for investigating long-term variations in Chl*a* at large scales.

As the third-largest freshwater lake in China, Lake Taihu has a water surface area of 2,338 km^2^ and a mean water depth of 1.9 m^9^. This large, shallow lake is located in one of the world’s most heavily populated regions, which has experienced rapid economic development in recent years (the Yangtze River delta region). The lake supplies water to the approximately 10 million residents of surrounding cities including Wuxi, Suzhou, and Huzhou^9^. Thus, Lake Taihu’s water quality is vital to local human activities and needs such as drinking, tourism, fishing, and shipping. It also plays a key role in regional ecosystem functioning^22^. Lake Taihu has become progressively more eutrophic since the 1980 s due to dramatic increases in nutrient loading from urban and agricultural development in its watershed, leading to the frequent formation of cyanobacterial blooms in the spring and summer^7^,^22^. Moreover, multi-annual warming trends have worsened these cyanobacterial blooms^17^. For example, the spring of 2007 cyanobacterial bloom event occurring in this lake caused the contamination of tap water and spurred a drinking water crisis in the city of Wuxi, directly affecting several million people^9^,^22^. In our previous study^20^, we proposed a spectral index derived from the first two bands of MODIS data that can be used to reflect Chl*a* information and to obtain long-term Chl*a* records from MODIS data for Lake Taihu. However, it should be noted that our previous study did not fully develop a Chl*a* estimation model and that only a simple relationship was found between the spectral index and Chl*a*; this is attributable to the fact that the main aim of that study^20^ was to estimate microcystin concentrations by means of Chl*a* values. Thus, the objectives of the present study are: (1) to complete the development of the MODIS Chl*a* estimation algorithm initially proposed through our previous study^20^, (2) to generate a long-term Chl*a* record based on MODIS-Aqua observations to help characterize long-term variability trends of cyanobacterial biomass (Chl*a*) and blooms in Lake Taihu, and (3) to determine how cyanobacteria respond to meteorological factors and nutrient variability levels by examining long-term Chl*a* records from MODIS-Aqua data.

## Materials and Methods

### Description of sampling sites

Field observations of nutrients including total nitrogen (TN) and phosphorus (TP) and of Chl*a* were collected monthly from pre-defined sites across Lake Taihu from 2003 to 2013. Eleven years of continuous monthly sampling at these sampling sites yielded a total of 2,432 water samples. The datasets were collected as part of a long-term monitoring project managed by the Taihu Laboratory for Lake Ecosystem Research (TLLER) of the Nanjing Institute of Geography and Limnology at the Chinese Academy of Sciences. From each sampling site, surface water samples were collected at four water depths (10 cm, 50 cm, 100 cm, and 150 cm), were added to 2 L acid-washed bottles and were then stored on ice while working in the field. All samples were transported to the TLLER on the day of collection. The samples included a broad range of biogeochemical and optical variability levels for Lake Taihu, including water samples with optical properties dominated by strong terrestrial inputs to samples made during strong cyanobacterial bloom events. Further information on the spatial distribution of the sampling sites in Lake Taihu can be found in our previous paper^20^. To better understand spatial variations in cyanobacterial biomass levels in aquatic environments of Lake Taihu, we divided the lake into six parts: Meiliang Bay, Zhushan Bay, Gonghu Bay, open area, Xukou Bay, and East Lake Taihu (Figure S1).

### Measurements of Chla, TN and TP concentrations

We recorded three water quality parameters: Chl*a*, TP, and TN. We used Whatman GF/F fiberglass filters with an average pore size of 0.7 μm to collect algal particles from the water samples. Chl*a* pigments were extracted using 90% ethanol at 80 °C and then were spectrophotometrically analyzed to measure their absorption coefficients at 665 nm and 750 nm. We then calculated Chl*a* concentrations from the absorption coefficients at those two wavelengths^41^. TP and TN concentrations were measured via combined persulfate digestion^42^. We fixed phytoplankton samples with Lugol’s iodine solution and sedimented them for 48 h prior to counting them using a microscope.

### Image data processing and FAI (Floating algae index) products

The MODIS-Aqua data have been freely available since 2002, and they have a maximum spatial resolution of 250 m (bands 1 and 2) and a very short revisit interval (1 image/day). MODIS-Aqua L-0 data for January of 2003 to of December 2013 (more than 4,000 images) were downloaded from NASA’s Goddard Space Flight Center website (GSFC, http://oceancolor.gsfc.nasa.gov/). As the daily MODIS-Aqua images often included clouds, cloud shadows, or thick aerosols, not all of the images downloaded were used in this study. We selected 1,109 cloud-free images of Lake Taihu for January of 2003 to December of 2013. These images were processed to Level-1 (calibrated spectral radiance) using the SeaDAS software package (version 6.0). Corrections for ozone, water vapor absorption, and molecular (Rayleigh) scattering were then performed following methods proposed through previous studies^7^, and FAI values were calculated for recognized and delineated floating algae in Lake Taihu using the methods of Hu *et al*. (2010b) (i.e., FAI > −0.004)^7^.

### Comparisons between MODIS-Aqua data and *in situ* measurements

To minimize effects of the temporal difference between the field and MODIS-Aqua data, we created a criterion for matching satellite and *in situ* data to ≤3 hours (the time interval between *in situ* and corresponding MODIS-Aqua measurements). Our criterion yielded 250 matched pairs of Rayleigh-corrected *R*_rc_ and *in situ* Chl*a* measurements; for these data pairs, we also required that the MODIS images and *in situ* data were located at the same points in space (the same pixel). The matching samples were distributed across the entire lake and across four seasons, thus representing an overall relationship between MODIS-Aqua and *in situ* Chl*a* measurements for Lake Taihu. The 250 *in situ* Chl*a-*Rayleigh-corrected *R*_rc_ data pairs were used to develop and validate the Chl*a* estimation model. First, we used half (125) of the 250 *in situ* Chl*a*-*R*_rc_ data pairs for model development and the other half for model validation. We randomly and evenly divided the 250 *in situ* Chl*a*-*R*_rc_ data pairs into two parts ten times. As a result, we produced nine groups of dataset pairs (125–125) to test the stability of the developed model. One group of dataset pairs was created for model development and validation and other nine groups of dataset pairs were used to test stability of the developed model. For this study, we developed an empirical model for the retrieval of Chl*a* from MODIS-Aqua data for Lake Taihu.

### Meteorological data

Daily wind speed (m/s), temperature (°C), and atmospheric pressure (hpa) data for 1956 to 2013 were obtained from the Dongshan meteorological station (31°4′, 120°26′E) and were downloaded from the China Meteorological Data Sharing Service System (http://cdc.nmic.cn).

### Statistical analysis and accuracy assessment

Statistical Program for Social Sciences (SPSS 17.0) software (version 17.0) was used to perform the statistical analyses. Pearson’s correlation analysis method was used to investigate relationships between the variables. Significance levels are reported as significant (*p* < 0.05) or not significant (*p* > 0.05).

We assessed the accuracy of the model developed using relative error (*RE*), mean absolute percent error (*MAPE*), and root-mean-square error (*RMSE*) values between the measured and predicted values using the following equations:


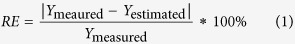



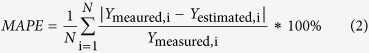



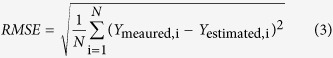


where N is the number of samples, and *Y*_measured_ and *Y*_estimated_ are the measured and estimated values, respectively.

CARTs (Classification and regression trees) were used to examine relationships between cyanobacterial dynamics and meteorological and nutrient factors to investigate the relative importance of various factors in controlling cyanobacterial dynamics. CARTs can explain the variability in a single dependent variable corresponding to one or more explanatory variables by splitting data recursively based on the most influential independent variable^43^. CARTs, as efficient tools for extracting key variables and thresholds from a multivariate dataset, have been widely used in a variety of fields such as environmental studies and ecology^43^. CART analyses were performed in this study using SPSS 17.0 software.

## Results

### Model development and validation

**Figure 1:**
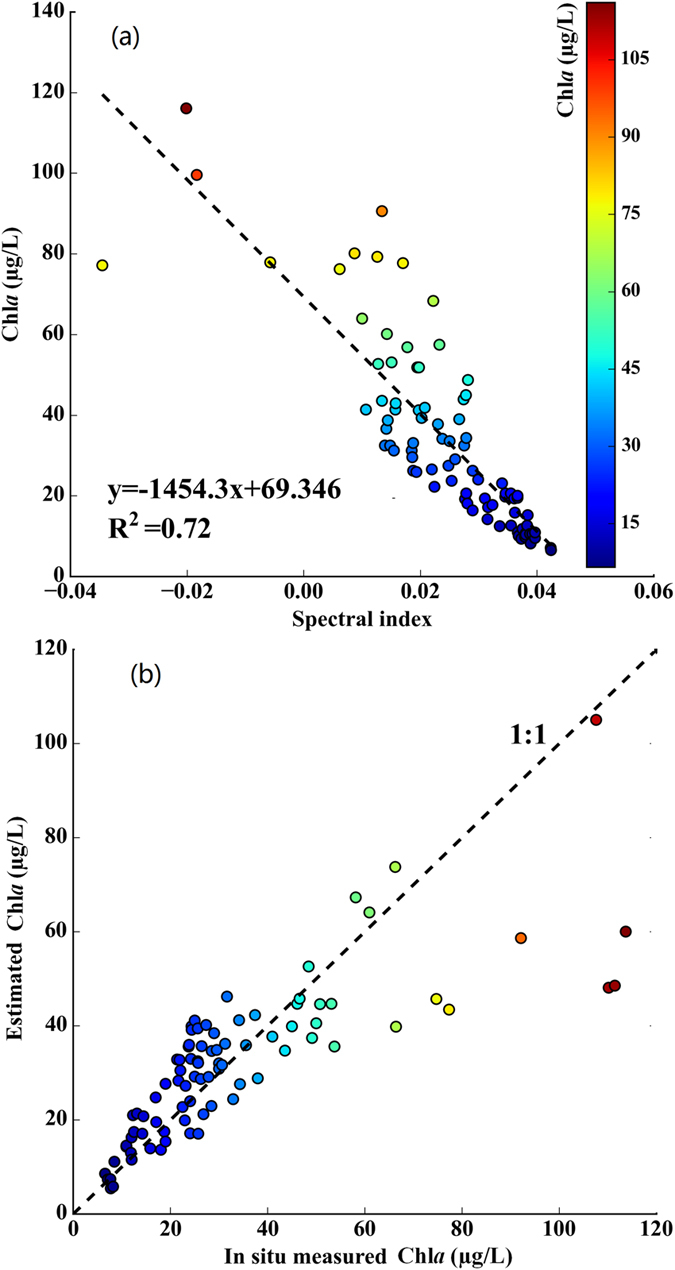
Calibration (**a**) and validation (**b**) of the proposed model for estimating Chl*a* in Lake Taihu. *R*_rc_ spectral index = (Exp(*R*_rc_(645)) − Exp(*R*_rc_(859)))/(Exp(*R*_rc_(645)) + Exp(*R*_rc_(859))).

The spectral index [(EXP(*R*_rc_(645)) − EXP(*R*_rc_(859)))/(EXP(*R*_rc_(645)) + EXP(*R*_rc_(859)))] proposed through our previous study was used to construct the Chl*a* estimation model^20^. We used linear, logarithmic, exponential, and quadratic functions to model *in situ* Chl*a* and the spectral index. Among these functions, the linear function offered the highest degree of modeling precision, the highest correlation coefficient (*r* = −0.85), and the lowest *MAPE* (24%) and *RMSE* (12.44 μg/L) values (Fig. 1(a)); the linear function is as follows:


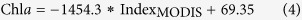


where Index_MODIS_ = [(EXP(*R*_rc_(645)) − EXP(*R*_rc_(859)))/(EXP(*R*_rc_(645)) + EXP(*R*_rc_(859)))], and *R*_rc_(645) and *R*_rc_(859) are atmospherically Rayleigh-corrected MODIS-Aqua data at 645 nm and 859 nm, respectively.

To assess the performance of the proposed Chl*a* estimation model, we used independent validation data from 125 matched *in situ* Chl*a*-*R*_rc_ data pairs (Chl*a*: 6.56–113.66 μg/L). Without adjusting the spectral index and re-parameterization, the proposed model generally performed well for Chl*a* estimations (Fig. 1(b)). The *RE* of the model for the validation dataset ranged from 0.4% to 64.5% with a *MAPE* of 27.1% (*RMSE* = 15.01 μg/L). The *RE* values of 40% and 60% of the samples fell below 20% and 30%, respectively. The *in situ* Chl*a* measurements and Chl*a* values estimated using the proposed model with the normalized spectral index [(EXP(*R*_rc_(645)) − EXP(*R*_rc_(859)))/(EXP(*R*_rc_(645)) + EXP(*R*_rc_(859)))] showed good agreement and a significant linear correlation (*p* < 0.005; *t*-test). In addition, the measured and estimated Chl*a* values were evenly distributed along a 1:1 line (Fig. 1(b)). These results suggest that the model developed from the normalized spectral index performs satisfactorily and can be used to derive Chl*a*. We separately used the remaining nine groups of dataset pairs to develop Chl*a* estimation models and to validate the developed model. We found no significant differences in the slopes and intercepts of the nine linear regression equations. The slopes and intercepts varied from −1,450.1 to −1,457.8 and from 53.11 to 72.86, respectively. *MAPE* values of the nine models for the corresponding validation datasets ranged from 25.5% to 30.6%. This suggests that the developed model is very stable. The model can thus be used to quantify spatial and temporal Chl*a* distributions for Lake Taihu.

### Cyanobacterial dynamics

Two indices of Chl*a* and the area of cyanobacterial surface blooms were used to characterize long-term trends in the cyanobacterial dynamics of Lake Taihu. The proposed model (Equation (4)) and the FAI proposed by Hu *et al*. (2010b)^7^ were used, respectively, to derive a Chl*a* time series and the cyanobacterial surface bloom areas for this lake from all available MODIS-Aqua data collected from January of 2003 to December of 2013. Several sub-regions of Lake Taihu, including Gonghu Bay, Xukou Bay, and East Lake Taihu, are known to be covered in aquatic plants such as weeds, reeds, and other macrophytes. These aquatic plants can significantly change the reflectance characteristics of a lake’s surface, and thus Chl*a* data and cyanobacterial bloom areas cannot be accurately derived from remote optical satellite data in such areas^20^. Thus, in the following, an “entire lake” refers to the entire area of Lake Taihu with the exception of these three areas (Gonghu Bay, Xukou Bay, and East Lake Taihu). Chl*a* images of yearly, monthly, and seasonal means for Lake Taihu were developed using the arithmetic means of all MODIS-derived Chl*a* products for between 2003 and 2013.

**Figure 2:**
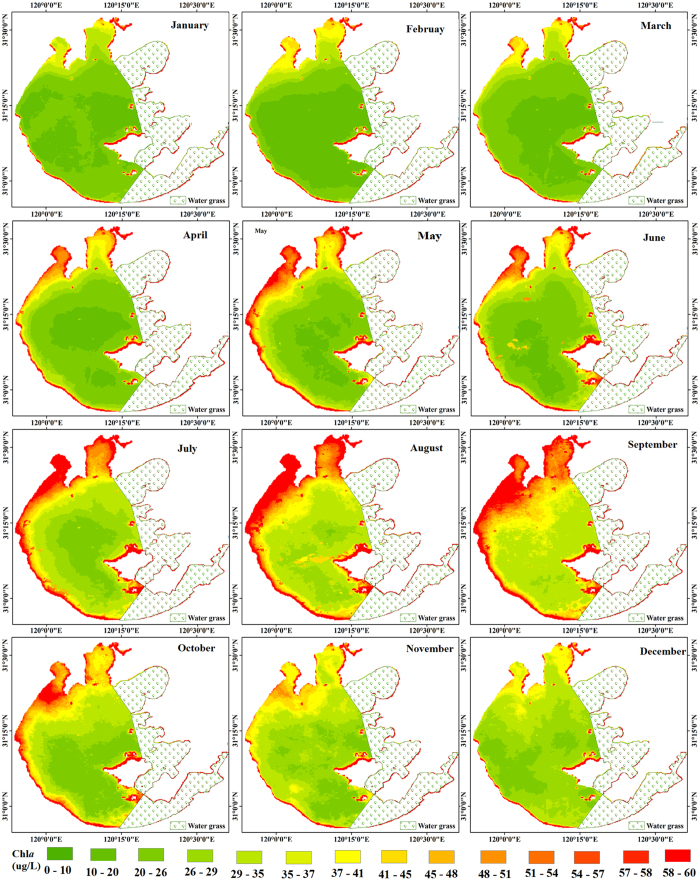
MODIS-Aqua derived (2003–2013) monthly mean Chl*a* for Lake Taihu for January to December. The figure was derived from MODIS-Aqua data using ENVI 5.0 (2013) (https://www.harris.com/solution/envi).

**Figure 3:**
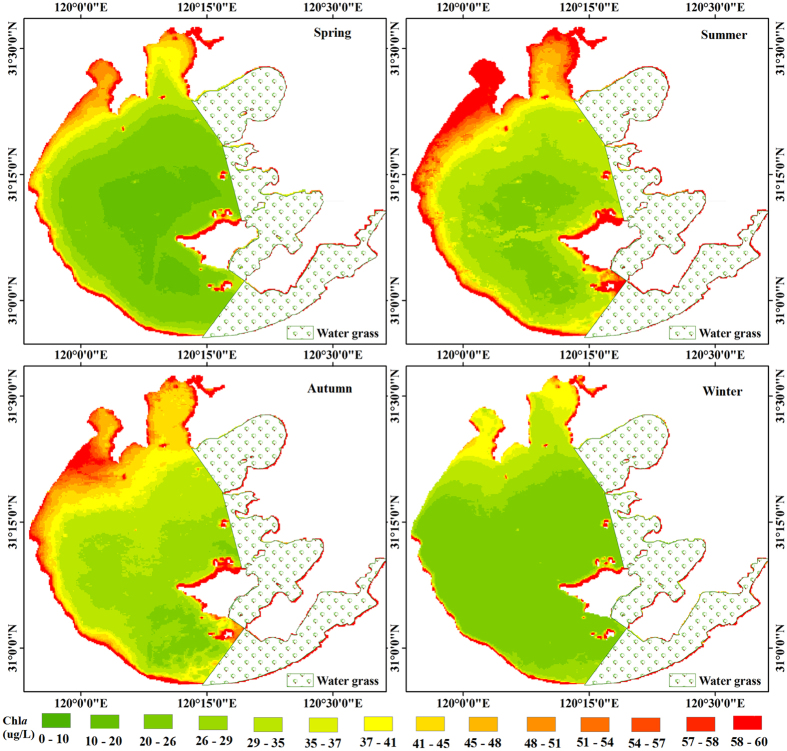
Maps of the MODIS-Aqua derived Chl*a* for all four seasons in Lake Taihu generated using the proposed model. The figure was derived from MODIS-Aqua data using ENVI 5.0 (2013) (https://www.harris.com/solution/envi).

Overall, the lake water shows relatively high Chl*a* values during all seasons. Chl*a* exhibits strong seasonal variability across the entire lake (Figs 2 and 3). Overall, Chl*a* is substantially higher during the summer (June-August) and autumn (September-November) than in the spring (March-May) and winter (December-February) (*p* < 0.005; *t*-test).

Mean seasonal Chl*a* values of the entire lake for spring to winter are 27.86 μg/L (standard deviation (SD) = 4.43 μg/L), 38.07 μg/L (SD = 3.81 μg/L), 37.32 μg/L (SD = 3.68 μg/L), and 27.05 μg/L (SD = 2.43 μg/L), respectively. The highest monthly mean Chl*a* value for the entire lake (42.67 μg/L) was reached in August and the lowest monthly mean Chl*a* value (22.92 μg/L) was reached in February. The highest and the lowest monthly mean Chl*a* values for both Meiliang Bay (with highest and lowest values of 58.42 μg/L and 33.04 μg/L, respectively) and Zhushan Bay (with highest and lowest values of 72.05 μg/L and 36.03 μg/L, respectively) were reached in August and January, respectively. In contrast, for open areas, the highest (September) and lowest (February) monthly mean Chl*a* values (with highest and lowest values of 21.12 μg/L and 40.81 μg/L, respectively) were reached one month later than those of the other two areas.

**Figure 4:**
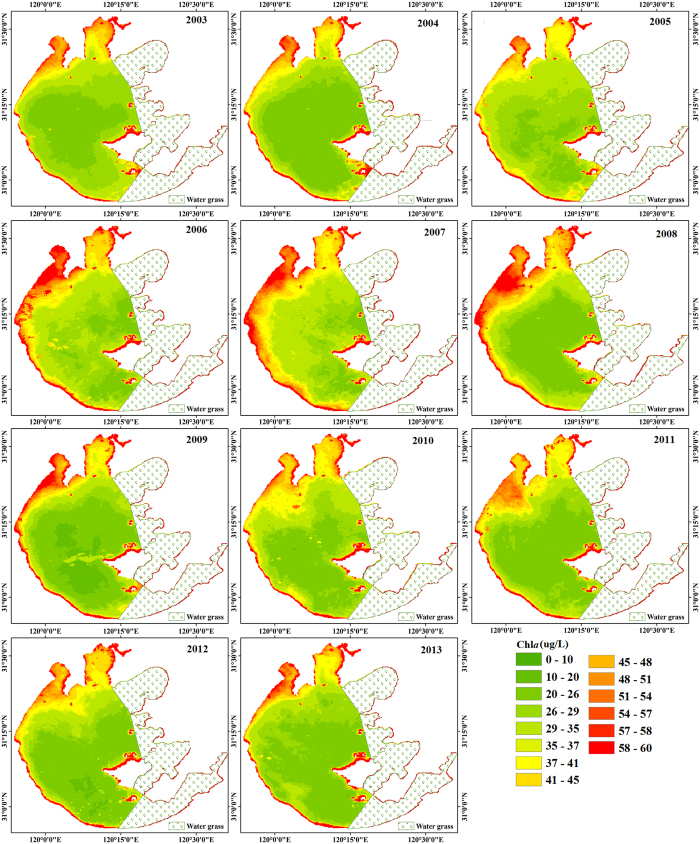
Annual mean Chl*a* distributions of Lake Taihu for 2003 to 2013. The figure was derived from MODIS-Aqua data using ENVI 5.0 (2013) (https://www.harris.com/solution/envi).

Substantial interannual variability in Chl*a* from 2003 to 2013 was found (Fig. 4). The lowest annual mean Chl*a* value for the entire lake was 30.81 μg/L in 2009, and the highest Chl*a* value reached was 37.28 μg/L, which occurred in 2007 (Fig. 4).

Generally, Chl*a* dynamics in Lake Taihu were characterized by three phases from 2003 to 2013. Chl*a* values increased sharply from 2003 to 2007 at a rate of 2.03 μg/L/year, after which they decreased from 2007 to 2009 (at a rate of 3.2 μg/L/year) and then increased slightly from 2009 to 2013.

**Figure 5:**
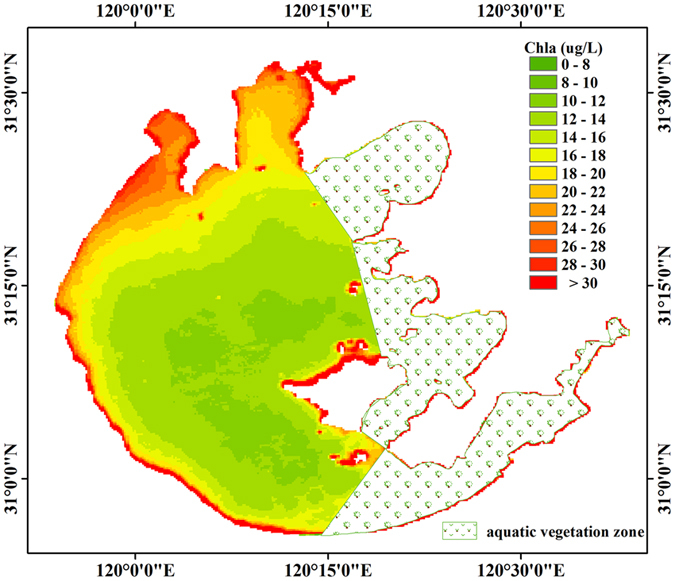
Chl*a* spatial distribution for Lake Taihu averaged from all Chl*a* estimates based on MODIS-Aqua data gathered from 2003–2013. The figure was derived from MODIS-Aqua data using ENVI 5.0 (2013) (https://www.harris.com/solution/envi).

Spatially, Chl*a* values of Zhushan Bay were higher than those of the other two sub-regions, and the lowest Chl*a* value was reached in the middle of the open areas (Fig. 5). In Meiliang Bay and Zhushan Bay, Chl*a* values decreased from the inner to the outer parts of the regions (Fig. 5).

**Figure 6:**
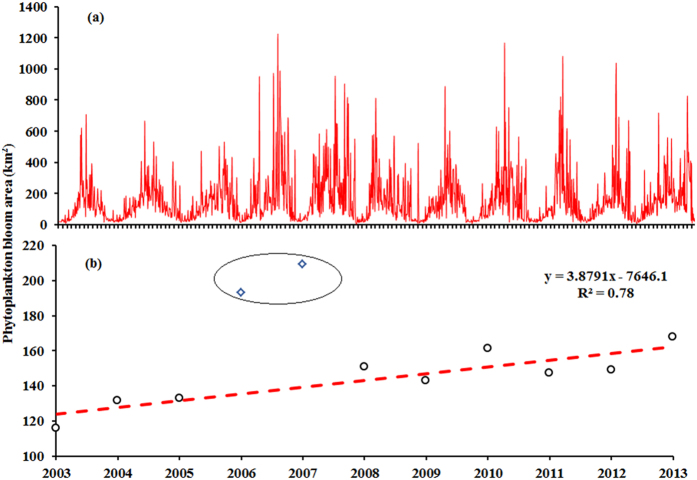
Monthly (**a**) and inter-annual (**b**) variations in cyanobacterial surface bloom areas in Lake Taihu from 2003 to 2013. Data for 2006 and 2007 (marked by the circle) were excluded when performing linear regressions between the bloom area and year.

For cyanobacterial surface bloom areas, a clear seasonal cycle was observed in Lake Taihu (Fig. 6(a)) whereby cyanobacterial blooms occurred much more often during the summer and autumn and less frequently in the spring and winter. Cyanobacterial surface bloom areas were also significantly higher in the summer and autumn than in the spring and winter (*p* < 0.005; *t*-test) (Fig. 6(a)).

We also found substantial interannual variations in the cyanobacterial surface bloom areas (Fig. 6(b)). For the entire lake, we found an obvious difference between 2006 and 2007 and other years. In 2006 and 2007, mean cyanobacterial surface bloom areas were larger than those existing during the other periods. For example, the mean area reached in 2007 was almost twice that reached in 2003. Cyanobacterial surface bloom areas were relatively small between 2003 and 2005, and the areas were larger than those of 2003 to 2005 from 2008 to 2013 but smaller than those of 2006 to 2007. In addition, the annual mean cyanobacterial surface bloom area expanded from 2003 to 2013 (R^2^ = 0.78, *p* < 0.005, excluding 2006 and 2007). The annual mean cyanobacterial surface bloom area of Lake Taihu gradually expanded from 115.91 km^2^ in 2003 to 167.77 km^2^ in 2013.

### Responses to nutrient enrichment and meteorological factors

**Figure 7:**
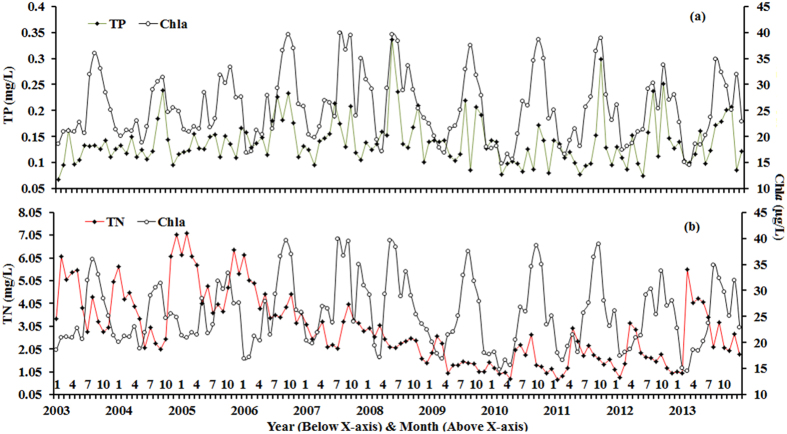
Monthly mean MODIS-Aqua derived Chl*a* and *in situ* measured TP and TN for 2003 to 2013.

The Chl*a* and TP time series are somewhat synchronous, showing similar variations except during the period of each year showing low Chl*a* and TP values (Fig. 7(a)). This indicates that Chl*a* responds differently to TP by season. The highest correlation coefficient (*r* = 0.91; p < 0.005) between Chl*a* and TP occurred in the spring with a simple linear function (Chl*a* = 89.91*TP + 10.14). In contrast, the two parameters were not significantly correlated during the other seasons. However, we found no consistent variation trend and no obvious relationship between Chl*a* and TN (Fig. 7(a))

These results suggest that Chl*a* is only phosphorus-limited in the spring, and they explain variations in Lake Taihu’s spring Chl*a* values. However, the other three seasons showed a weak relationship between Chl*a* and TP, meaning that Chl*a* is not phosphorus-limited during these seasons.

**Figure 8:**
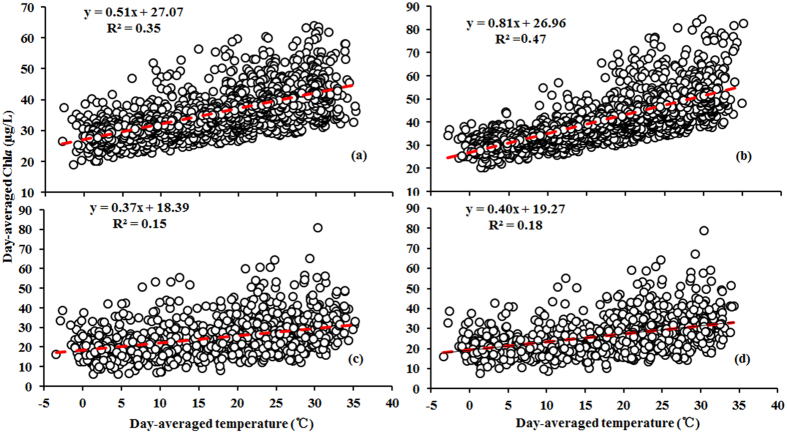
Relationships between daily mean Chl*a* and air temperature in Meiliang Bay (**a**), Zhushan Bay (**b**), open areas (**c**), and the entire lake area (**d**) from 2003 to 2013.

Significantly positive correlations were found between the daily mean MODIS-Aqua derived Chl*a* and air temperature for Lake Taihu (*r* = 0.43, *p* < 0.005) (Fig. 8). We note that Pearson’s correlation coefficients between Chl*a* and temperature vary by sub-region: Pearson’s correlation coefficients for Zhushan Bay (*r* = 0.69, *p* < 0.005) and Meiliang Bay (*r* = 0.60, *p* < 0.005) are higher than those of the open areas (*r* = 0.39, *p* < 0.005).

No significant correlation was found between daily mean MODIS-Aqua derived Chl*a* and wind speed. This is likely attributed to other meteorological factors, such as air temperature, that drive the majority of daily variations in cyanobacterial biomass. Previous studies have suggested that wind has spatially variable effects on Lake Taihu^44^,^45^, indicating that wind may affect Chl*a* spatial variations independently of other meteorological effects. The spatial relationship between wind effects and Chl*a* for this lake was not investigated, as we did not have access to the spatial distribution of wind fields across Lake Taihu.

Overall, our results suggest that air temperature plays a critical role in the cyanobacterial dynamics of Lake Taihu. More specifically, high air temperatures and low pressure levels prompt cyanobacterial bloom formation. The effects of air temperature and pressure vary depending on the sub-region of Lake Taihu concerned; these variables have a stronger impact on cyanobacterial growth in Zhushan Bay and Meiliang Bay, where wind and wave effects are relatively weak and where nutrient levels are high. However, in open areas with strong wind effects and low nutrient levels, effects of air temperature on cyanobacterial growth are less pronounced. Thus, our results suggest that it is necessary to differentiate between the various sub-regions of Lake Taihu when studying the effects of air temperature and pressure on cyanobacterial growth.

**Figure 9:**
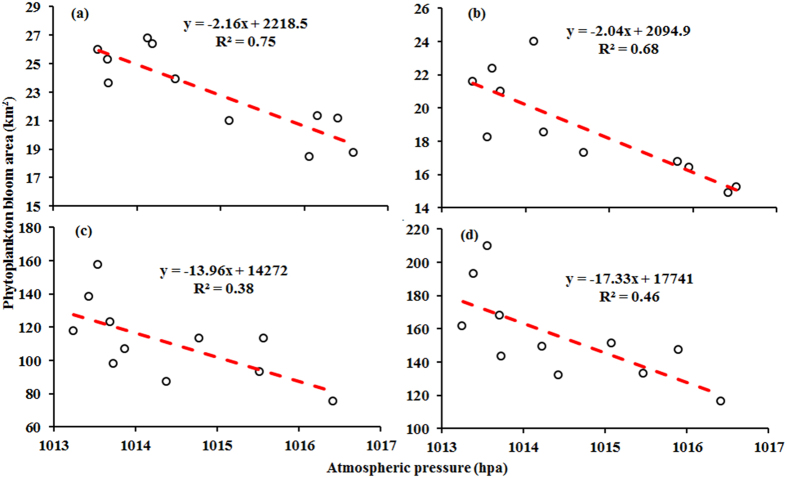
Relationships between annual mean cyanobacterial surface bloom areas and atmospheric pressure in Meiliang Bay (**a**), in Zhushan Bay (**b**), in open areas (**c**), and across the entire lake (**d**) for 2003 to 2013.

The Pearson’s correlation coefficient between the two parameters was found to be highest in Meiliang Bay (*r* = 0.87, *p* < 0.0005) and Zhushan Bay (*r* = 0.82, *p* < 0.0005) and lower in open areas (*r* = 0.62, *p* < 0.0005). Across the entire lake area, there is a significantly negative correlation between annual cyanobacterial surface bloom areas and atmospheric pressure levels (*r* = 0.68, *p* < 0.0005) (Fig. 9), suggesting that cyanobacterial blooms are more likely to occur during periods of low atmospheric pressure in Lake Taihu.

**Figure 10:**
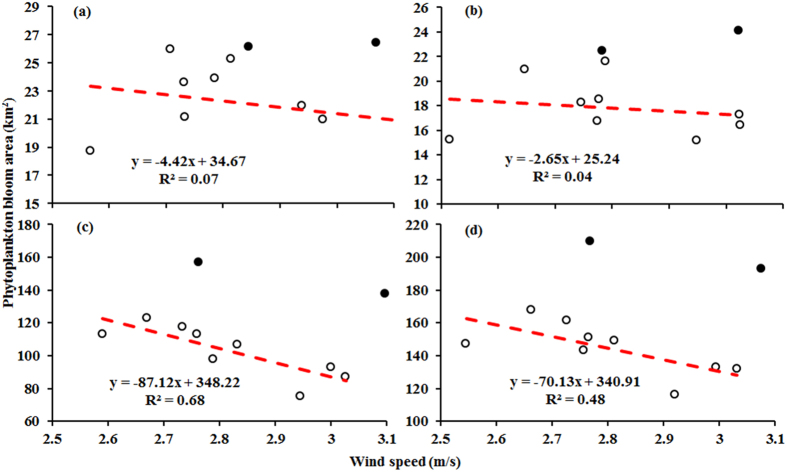
Relationships between annual mean cyanobacterial surface bloom areas and wind speed for Meiliang Bay (**a**), Zhushan Bay (**b**), open areas (**c**), and the entire lake (**d**) from 2003 to 2013. Note that we exclude data for 2006 and 2007, denoted by solid black dots in the figure.

Overall, annual mean cyanobacterial surface bloom areas contracted as wind speeds increased (Fig. 10). However, no significant correlation was found between annual mean cyanobacterial surface bloom areas and wind speed. When we exclude the data for 2006 and 2007, which include anomalously large bloom areas, we find statistically significant negative correlations for Lake Taihu (from 2003 to 2005 and from 2008 to 2013) (*r* = −0.69, *p* < 0.0005) and especially for open areas (*r* = −0.82, *p* < 0.0005) (Fig. 10). These correlations indicate that low wind speeds favor cyanobacterial surface bloom occurrence. In comparison, correlation coefficients for Meiliang Bay (*r* = −0.69, *p* < 0.0005) and Zhushan Bay were found to be lower than those of open areas, indicating that cyanobacterial blooms are less sensitive to wind speed in the two bays than in the open areas.

Our results suggest that low atmospheric pressure and wind speed levels can be hypothesized to facilitate cyanobacterial surface bloom formation. Cyanobacterial blooms in Lake Taihu are generally hindered by high atmospheric pressure and strong winds. However, the responses of cyanobacterial blooms in Lake Taihu to atmospheric pressure and wind speed vary spatially. Cyanobacterial blooms are more sensitive to atmospheric pressure in Meiliang Bay and Zhushan Bay than in open areas; however, wind speed more heavily controls cyanobacterial blooms in open areas than in the two bays.

### The effects of nutrient enrichment and meteorological factors on cyanobacterial dynamics

As relationships between cyanobacterial biomass and these environmental factors may be non-linear and may involve complex interactions, we utilized the CART model to further examine effects of the environmental factors on inter-annual Chl*a* and cyanobacterial surface bloom area dynamics. Chl*a* was found to be sensitive to TP and temperature, but cyanobacterial surface bloom areas were found to be sensitive to air pressure and wind speed. Therefore, TP and temperature levels were used as Chl*a* CART model inputs while air pressure and wind speed values were used in the cyanobacterial surface bloom CART model.

**Figure 11:**
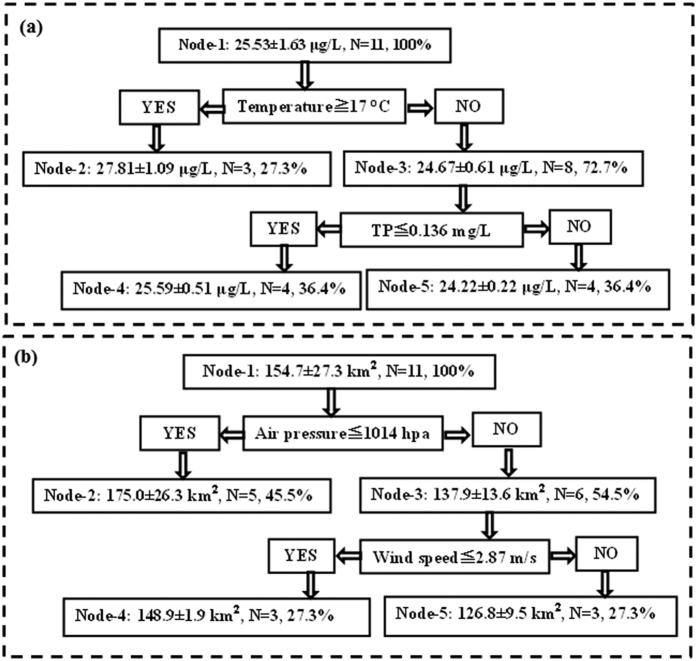
CART models for Chl*a* (**a**) and cyanobacterial surface bloom area values (**b**).

A root node (Node-1) that included all of the data (N = 11 years, data = 100%) was used as a starting point (Fig. 11(a)). Temperature was found to be the most important factor affecting inter-annual variability in Chl*a* values in Lake Taihu. The average Chl*a* value for this group was 25.53 μg/L (SD = 1.63 μg/L). The observations were divided (threshold value = 17 °C) further based on high and low temperatures. Under higher temperatures, relatively higher Chl*a* values were observed in Lake Taihu (node = 2, mean = 27.81 μg/L (SD = 1.09 μg/L), percentage = 27.3%). Under lower temperatures, TP values also heavily affected Chl*a* levels in Lake Taihu. A significant increase in TP (≧0.136 mg/L) produced high Chl*a* conditions (node = 5, mean = 25.59 μg/L (SD = 0.51 μg/L), percentage = 36.3%) at this node. In summary, high temperature, high TP and low TP values were found to control inter-annual variations in Chl*a* by approximately 27.3%, 36.4% and 36.4%, respectively, in Lake Taihu from 2003 to 2013. These results indicate that temperature was the most important factor affecting inter-annual Chl*a* variability (followed by TP) in Lake Taihu from 2003 to 2013.

A root node (Node-1) that included all of the data (N = 11 years, data = 100%) was used as a starting point (Fig. 11(b)). Air pressure was found to be the most important factor affecting the inter-annual dynamics of cyanobacterial bloom areas in Lake Taihu. The average cyanobacterial surface bloom area for this group was 154.7 km^2^ (SD = 27.3 km^2^). Observations were divided (threshold value = 1,014 hpa) further based on high and low air pressure values. Under lower air pressure conditions, relatively larger cyanobacterial surface bloom areas were observed in Lake Taihu (node = 2, mean = 175.0 km^2^ (SD = 26.3 km^2^), percentage = 45.5%). Under higher air pressure conditions, wind speeds also heavily affected the formation of cyanobacterial surface blooms in Lake Taihu. A significant decrease in wind speed (≦2.87 m/s) expanded the cyanobacterial surface bloom area (node = 4, mean = 148.9 km^2^ (SD = 1.9 km^2^), percentage = 27.3%) at this node. In summary, low air pressure, low wind speed and high wind speed values controlled inter-annual variations in cyanobacterial surface bloom area values by approximately 45.5%, 27.3% and 27.2%, respectively, in Lake Taihu from 2003 to 2013. These results indicate that air pressure was the most important factor controlling cyanobacterial surface bloom formation (followed by wind speed) in Lake Taihu from 2003 to 2013.

## Discussion

### Advantages of the 11-year MODIS Cyanobacteria data records

Lake Taihu is managed through an extensive water quality observational network based on a strict nutrient management strategy. However, monthly field sampling can introduce uncertainties into both short- and long-term observations of water quality (e.g., Chl*a*) dynamics due to rapid changes that occur in this lake^45^. In addition, Lake Taihu is subject to complex interacting dynamics and often experiences changes in aquatic system functioning due to various physical, chemical and biotic drivers^9^. All of these factors make it necessary to develop a remote sensing approach for monitoring Lake Taihu’s spatial and temporal dynamics in water quality and to quantify its responses to environmental drivers.

Recently, Qi *et al*. (2014) developed and validated a novel algorithm based on MERIS data for estimating phycocyanin concentrations of cyanobacteria in Lake Taihu^46^. Using the model and 512 MERIS images, long-term records of phycocyanin data were constructed through their study, which generated valuable information for the study of cyanobacterial biomass dynamics. However, our studies offer more benefits than those of Qi *et al*. (2014). First, our consideration of many more calibration and validation datasets (250 data pairs) covering the entire area of Lake Taihu and all four seasons ensures the reliability of our Chl*a* estimation model for Lake Taihu. Second, we considered many more high-quality MODIS images (1,109 images) relative to the number of MERIS images collected in Qi *et al*. (2014) (512 images), and the use of more long-term Chl*a* data should generate more reliable results overall. Second, though MERIS services have be available from April of 2012, MODIS data can be used to continually to monitor Chl*a* in the future.

Based on field observations and laboratory experiments, previous studies have suggested that the frequent cyanobacterial blooms occurring in Lake Taihu have resulted from a combination of environmental factors, including nutrient inputs and climatic conditions^9^,^22^,^23^,^46^,^47^,^48^. However, the results of these studies have largely been based on samples drawn from a limited number of sites and over short time intervals, and so their conclusions are based on data of low spatial and temporal resolutions. It is well known that Lake Taihu’s biogeochemical parameters are characterized by complex spatial and temporal dynamics^10^,^22^.

Paerl *et al*.^49^ used a series of nutrient addition bioassays to determine that cyanobacterial growth in Lake Taihu is co-limited by nitrogen and phosphorus^49^; their study also showed that nitrogen and phosphorus limitations change seasonally, with phosphorus limitations generally occurring in the early spring and nitrogen limitations occurring from the summer to autumn (Paerl *et al*.)^49^. Similar results on nutrient limitations in Lake Taihu have been drawn by Xu *et al*.^29^ using the same approach as that of Paerl *et al*.^49^. Clearly, we appear to present results contradictory to those of these previous studies^29^,^50^ regarding whether nitrogen limits cyanobacterial growth. The differing time and spatial scales of data used in our study and previous studies^45^,^49^ may have caused these differences. In determining whether nitrogen or phosphorus limits cyanobacterial growth, Xu *et al*.^29^ performed four *in situ* nutrient addition experiments in May, July, October, and December of 2008^29^. In the study (Xu *et al*.)^29^, each nutrient addition experiment lasted several days (day scale), and three sites in Lake Taihu were used for the four experiments (specific site scale)^29^. In our study, both Chl*a* and nutrient data were collected for 2003 to 2013 for the entire lake area. Our results are thus based on an examination of the entire lake area on an annual scale, thus revealing the longer-term responses of cyanobacterial dynamics to nutrients at a broader spatial scale.

The following points could explain the seasonality of our findings. Our long-term *in situ* measurements showed that the ratio values of TN:TP for all seasons from 2003 to 2013 are significantly higher than the Redfield ratio (16:1)^51^, which has been widely used to identify nitrogen limitations (when TP:TN < 16:1) for Lake Taihu. Second, the spring season is typically considered to be a season of cyanobacterial growth during which cyanobacteria multiply rapidly and require higher levels of phosphorus. In Lake Taihu, TP levels were found to be lower in the spring than during other seasons, when they were high enough not to limit cyanobacterial growth. In addition, ratios of TN:TP in the spring with an average value of 45:1 significantly exceed the Redfield ratio (16:1) (closed to 3 times the Redfield ratio (16:1)), revealing the reasonability of phosphorus limitations in the spring in Lake Taihu. Based on laboratory experiments and field observations, previous studies have suggested that temperature increases can prompt significant cyanobacterial growth^4^, can cause initial bloom times to occur earlier on^23^, and can prolong annual bloom periods^52^. These data, however, due to their inherent limitations, are discrete and sporadic in nature, thus preventing us from conducting a thorough and objective evaluation of climatic driven factors of cyanobacterial dynamics in Lake Taihu. By taking advantage of the MODIS cyanobacterial time series for 2003–2013 generated using a new developed model together with historical records of meteorological data, we offer a comprehensive understanding of the climatic forces driving such cyanobacterial changes. We found that temperature levels can prompt cyanobacterial growth and that cyanobacterial surface bloom patterns are sensitive to wind speeds. The sensitivity of these climatic factors to cyanobacterial dynamics varies regionally. We also made a hypothesis that air pressures may prompt cyanobacterial surface bloom formation in Lake Taihu. Atmospheric pressure has not traditionally been considered a significant factor shaping cyanobacterial surface bloom formation. To date, there are no previous studies that have linked atmospheric pressure to cyanobacterial surface bloom formation. Our results reveal a clear increase in cyanobacterial surface bloom area with a decrease in atmospheric pressure, suggesting that atmospheric pressure can be hypothesized to drive cyanobacterial bloom formation in Lake Taihu. The finding that atmospheric pressure plays a role in cyanobacterial bloom formation contributes a new perspective on the phenomenon of cyanobacterial blooms. Forecasting models for cyanobacterial blooms could benefit from including atmospheric pressure as a predicting parameter. Bubbles resulting from low atmospheric pressure should encourage surface cyanobacterial bloom formation^53^. Bubbles in water can result from the oversaturation of methane, carbon dioxide, nitrogen, or oxygen. Thus, as atmospheric pressure decreases, gas solubility in water is reduced, in turn leading to gas oversaturation and gas bubble growth.

### Implications for future lake management

**Figure 12:**
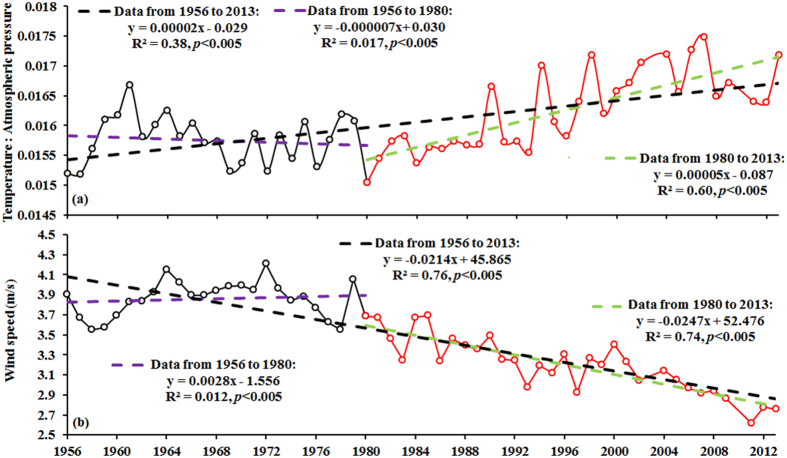
Annual mean ratio of air temperature to atmospheric pressure and wind speed observed at the Dongshan meteorological station from 1956 to 2013.

The effects of meteorological factors on cyanobacterial blooms necessitate a different interpretation of the phenomenon of cyanobacterial blooms. Forecasting models for cyanobacterial blooms could benefit from considering meteorological factors such as air temperature, atmospheric pressure, and wind as prediction parameters. It is noteworthy that from the 1980 s to the present, the ratio of air temperature to atmospheric pressure has significantly increased while wind patterns have declined in the Lake Taihu region (Fig. 12). However, these trends were not apparent from 1956 to 1980 (Fig. 12). Our results suggest that these trends in the ratio of air temperature to atmospheric pressure and wind speed could lead to the generation of more severe cyanobacterial blooms in Lake Taihu. Thus, severe cyanobacterial bloom events in Lake Taihu since the 1980 s could be attributed at least in part to changes in meteorological conditions. As climate change continues to affect the region, we can infer that the severity of cyanobacterial blooms will be enhanced in Lake Taihu.

The long-term Chl*a* data with high spatial and temporal resolutions used in this study are valuable in identifying the synergistic effects of nutrient loading on cyanobacterial growth. Our finding that Lake Taihu is only subject to phosphorus limitations in the spring has implications for controlling cyanobacterial blooms via the reduction of nutrients. This result highlights that severe cyanobacterial blooms may form during the spring seasons of years presenting high TP levels. It can also be inferred that phosphorus reduction during the spring may help control cyanobacterial blooms over short timescales; in contrast, the lack of Chl*a* sensitivity to TN found suggests that efforts to control the magnitude and duration of cyanobacterial blooms should not focus on nitrogen reduction in Lake Taihu. Furthermore, our results suggest that nutrient reduction strategies for controlling cyanobacterial blooms must take into account co-occurring climatic changes that favor cyanobacterial bloom development. More specifically, critical nutrient thresholds above which cyanobacterial blooms are prompted under favorable meteorological conditions (increases in temperature and decreases in atmospheric pressure and wind speed) should be reduced to compensate for more favorable growth conditions^50^. Moreover, the adjustment of nutrient reduction strategies should be regionally specific, as the sensitivity of cyanobacterial dynamics to these meteorological conditions varies across different sub-regions of Lake Taihu.

The results of this study have significant environmental implications according to the long-term monitoring of cyanobacterial dynamics in Lake Taihu. This is particularly critical in a changing climate, as it is often difficult to elucidate the causal factors of aquatic environmental changes without continuous and long-term water quality monitoring. Our results will help managers and decision-makers account for and modify their strategies for controlling cyanobacterial blooms in response to future climate change and human impacts. We thus recommend that remote sensing approaches be incorporated into future Lake Taihu management systems.

On a broader scale, the approaches and findings of this study may be extended to other lakes in which cyanobacteria dominate such as Lake Chaohu^54^, Lake Dianchi^50^, and Lake Erie. Once the Chl*a* estimation model is tuned with local data and once satellite-based Chl*a* are validated, similar long-term Chl*a* series analyses can be conducted with little effort and cost. Climatic warming and anthropogenically enhanced nutrient loading in lakes around the world are also known to be potential drivers of cyanobacterial surface bloom intensity levels^50^. The integration of satellite-derived data products with other *in situ* environmental and meteorological data will further aid in elucidating the cause and effect relationships between cyanobacterial dynamics and environmental and meteorological factors.

## Conclusions

We used remote sensing techniques to address this issue in Lake Taihu, which is a large, shallow and eutrophic lake. We first developed and validated an empirical model for estimating Chl*a* levels in Lake Taihu based on a spectral index derived from *R*_rc_ and *in situ* data. The proposed model exhibited robust performance for an independent validating dataset, with relative errors (*RE*) ranging from 0.4% to 64.5% with a mean absolute percent error (*MAPE*) value of 27.1% (*RMSE* = 15.01 μg/L) and from 6.56 μg/L to 113.66 μg/L for Chl*a*. Second, long-term Chl*a* and cyanobacterial surface bloom area time series were generated from MODIS-Aqua observations gathered from 2003 to 2013 by means of the proposed model and the floating algae index (FAI). Chl*a* values for Lake Taihu varied considerably in space and time, with higher Chl*a* values found for Zhushan Bay and Meiliang Bay and with lower Chl*a* values found for open areas. Chl*a* values in the summer and autumn were found to be significantly higher than those recorded in the spring and winter (*p* < 0.005). The annual mean area of cyanobacterial surface bloom events exhibited a clearly increasing trend for the entire lake area from 2003 to 2013 with the exception of 2006 and 2007 (annual mean areas = 3.8791*year-7646.1, *p* < 0.0005). From 2003 to 2013, the area gradually expanded from 115.91 km^2^ to 167.77 km^2^. Third, based on the long-term Chl*a* and cyanobacterial bloom area derived from MODIS-Aqua observations, we quantified the responses of cyanobacterial dynamics to nutrient enrichment and climatic conditions. The results show that high air temperatures and high phosphorus levels in the spring may prompt cyanobacterial growth and that low wind speeds and atmospheric pressure levels may favor cyanobacterial surface bloom formation. The sensitivity of cyanobacterial dynamics to climatic conditions was found to vary regionally, with more sensitivity to air temperature and atmospheric pressure observed in Zhushan Bay and Meiliang Bay and with more sensitivity to wind speed found in open areas.

## Additional Information

**How to cite this article**: Shi, K. *et al*. Long-term MODIS observations of cyanobacterial dynamics in Lake Taihu: Responses to nutrient enrichment and meteorological factors. *Sci. Rep.* **7**, 40326; doi: 10.1038/srep40326 (2017).

**Publisher's note:** Springer Nature remains neutral with regard to jurisdictional claims in published maps and institutional affiliations.

## Supplementary Material

Supplementary Information
