# Supplementary material for: Long-term MODIS observations of cyanobacterial dynamics in Lake Taihu: Responses to nutrient enrichment and meteorological factors
Source: Sci Rep. 2017 Jan 11;7:40326. doi: 10.1038/srep40326 (PMC5225474; doi:10.1038/srep40326)
Supplement: Supplementary Information [file srep40326-s1.pdf]

Long-term MODIS observations of cyanobacterial dynamics in Lake Taihu: Responses to nutrient enrichment and meteorological factors

Kun Shi[a, b], Yunlin Zhang[a*], Yongqiang Zhou[a, b], Xiaohan Liu[a, b], Guangwei Zhu[a], Boqiang Qin[a], Guang Gao[a]

[a] Taihu Laboratory for Lake Ecosystem Research, State Key Laboratory of Lake Science and Environment, Nanjing Institute of Geography and Limnology, Chinese Academy of Sciences, Nanjing 210008, China

[b] State Key Laboratory of Satellite Ocean Environment Dynamics（Second Institute of Oceanography, SOA

[c] University of Chinese Academy of Sciences, Beijing 100049, China

Supplementary Information

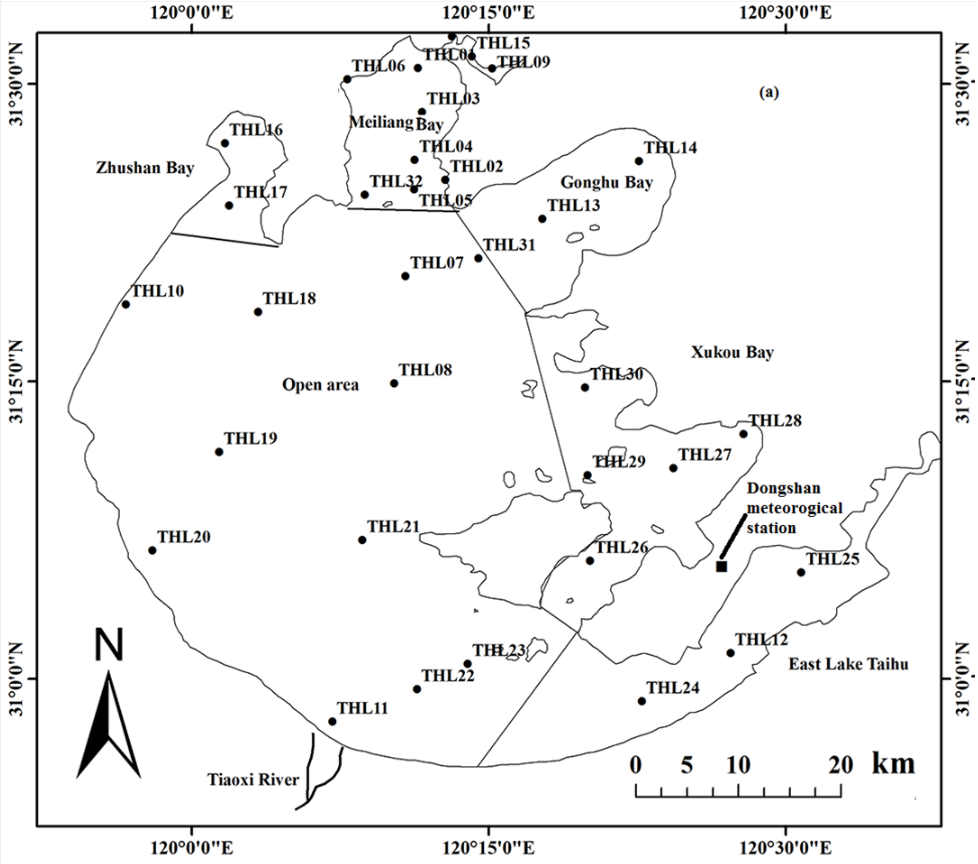

Figure S1 Distributions of sampling sites. (a) indicates the monthly cruises from 2003 to 2013; THL00–THL09 sites are for odd-numbered months from 2003–2004 and THL00–THL13 sites are for even-numbered months during these years; sites THL00–THL17 (except sites THL02, THL09, THL11–12, and THL15) are for the second two months of each season from 2005–2013, and sites THLL00–THLL32 (except THLL02) are for the last month of each season during these years. The figure created by Kun Shi with ArcGIS 10.2 (2013) (http://www.esri.com/software/arcgis)
